# The inhibition of NCS-1 binding to Ric8a rescues fragile X syndrome mice model phenotypes

**DOI:** 10.3389/fnins.2022.1007531

**Published:** 2022-11-16

**Authors:** Patricia Cogram, Luis C. Fernández-Beltrán, María José Casarejos, Sonia Sánchez-Yepes, Eulalia Rodríguez-Martín, Alfonso García-Rubia, María José Sánchez-Barrena, Carmen Gil, Ana Martínez, Alicia Mansilla

**Affiliations:** ^1^Department of Genetics, Institute of Ecology and Biodiversity (IEB), Faculty of Sciences, Universidad de Chile, Santiago, Chile; ^2^FRAXA-DVI, FRAXA Research Foundation, Santiago, Chile; ^3^Department of Neurobiology, Instituto Ramón y Cajal de Investigación Sanitaria, Hospital Universitario Ramón y Cajal, Madrid, Spain; ^4^Department of Immunology, Instituto Ramón y Cajal de Investigación Sanitaria, Hospital Universitario Ramón y Cajal, Madrid, Spain; ^5^Centro de Investigaciones Biológicas Margarita Salas-CSIC, Madrid, Spain; ^6^Centro de Investigación Biomédica en Red en Enfermedades Neurodegenerativas (CIBERNED), Instituto de Salud Carlos III, Madrid, Spain; ^7^Instituto de Química-Física Rocasolano, Spanish National Research Council, Madrid, Spain; ^8^Department of Biology Systems, Universidad de Alcala, Madrid, Spain

**Keywords:** Fragile X syndrome, Ncs-1, Ric8a, dopamine, protein-protein interaction inhibitor, Fmr1 knockout

## Abstract

Fragile X syndrome (FXS) is caused by the loss of function of Fragile X mental retardation protein (FMRP). FXS is one of the leading monogenic causes of intellectual disability (ID) and autism. Although it is caused by the failure of a single gene, FMRP that functions as an RNA binding protein affects a large number of genes secondarily. All these genes represent hundreds of potential targets and different mechanisms that account for multiple pathological features, thereby hampering the search for effective treatments. In this scenario, it seems desirable to reorient therapies toward more general approaches. Neuronal calcium sensor 1 (NCS-1), through its interaction with the guanine-exchange factor Ric8a, regulates the number of synapses and the probability of the release of a neurotransmitter, the two neuronal features that are altered in FXS and other neurodevelopmental disorders. Inhibitors of the NCS-1/Ric8a complex have been shown to be effective in restoring abnormally high synapse numbers as well as improving associative learning in FMRP mutant flies. Here, we demonstrate that phenothiazine FD44, an NCS-1/Ric8a inhibitor, has strong inhibition ability *in situ* and sufficient bioavailability in the mouse brain. More importantly, administration of FD44 to two different FXS mouse models restores well-known FXS phenotypes, such as hyperactivity, associative learning, aggressive behavior, stereotype, or impaired social approach. It has been suggested that dopamine (DA) may play a relevant role in the behavior and in neurodevelopmental disorders in general. We have measured DA and its metabolites in different brain regions, finding a higher metabolic rate in the limbic area, which is also restored with FD44 treatment. Therefore, in addition to confirming that the NCS-1/Ric8a complex is an excellent therapeutic target, we demonstrate the rescue effect of its inhibitor on the behavior of cognitive and autistic FXS mice and show DA metabolism as a FXS biochemical disease marker.

## Introduction

Autism spectrum disorders (ASDs) and intellectual disability (ID) are neurodevelopmental disorders that often present with variable and overlapping spectrum of symptoms (Cervantes and Matson, 2015; Dj and Ma, 2020). These conditions affect children from birth, particularly social interactions and communication skills, cognition, and adaptive behaviors (Thapar et al., 2017).

The etiology of 70% of all ASD/ID cases remains unknown due to the complex interplay of environmental and genetic factors (Chiurazzi et al., 2020). Gut microbiota composition (Kang et al., 2019) and environmental stress factors such as poor nutrition, infections, or socioeconomic causes may contribute to pathology (Bölte et al., 2019; Han et al., 2021). Genetic causality lies in polygenic risk involving small effect size variants in hundreds of genes. In fact, 1,400 human genes are described in Online Mendelian Inheritance in Man (OMIM) as being associated with ASD or ID or both. In contrast, there is a minority of cases that correspond to syndromic forms caused by high penetration single-gene mutations (Jeste and Geschwind, 2014; Vissers et al., 2016). Syndromic forms have proven to be of great help in constructing the pathophysiology of these disorders through a bottom-up approach (Cook et al., 2014; Ziats et al., 2021).

Fragile X syndrome (FXS) is the most common inherited cause of ASD and ID. It is caused by the loss of function of the fragile X mental retardation 1 (*fmr1*) gene (Verkerk et al., 1991), most commonly due to an unstable CGG-trinucleotide repeat that leads to its hypermethylation and transcriptional silencing (Sutcliffe et al., 1992). The *fmr1* product, Fragile X mental retardation protein (FMRP), is a messenger ribonucleic acid- (mRNA-) binding protein, which typically acts as a negative regulator of protein translation, facilitates mRNA trafficking, and influences mRNA stability (Zalfa et al., 2006). The failure in FXS is in a single gene, but due to its function, many other genes are secondarily affected, causing their RNAs to be underregulated and primarily causing protein overexpression (Darnell et al., 2011; Ascano et al., 2012). This overexpression is linked to deviations in the balance of excitatory vs. inhibitory circuits (Frye et al., 2016) and has been associated with increased spine density and decreased plasticity (Bagni and Zukin, 2019) in FXS.

Among FMRP target genes, metabotropic glutamate receptors (mGluR) and γ-aminobutyric acid (GABA) receptors are considered the most relevant in the etiopathogenesis of the disease (Bear et al., 2004; Braat and Kooy, 2015). However, despite some promising preclinical results with mGluR or GABA signaling modulators in FXS (Castagnola et al., 2017; Dahlhaus, 2018; Cogram et al., 2019), no strategy has so far provided successful therapeutic outcomes most likely because it just does not account for the full range of FXS abnormalities (Berry-Kravis et al., 2017, 2018). Here, we show a therapeutic approach that does not focus on any of the FMRP-regulated genes, but on a new strategy focused on rebalancing synaptic dysfunction. In fact, FXS and ASD have in common the dysregulation of synaptic density and maturation (Pfeiffer and Huber, 2009; Bagni and Zukin, 2019), leading to altered brain architecture and function (Hansel, 2019; Lee et al., 2019).

Normal neuronal function requires tight control of synapse homeostasis (Tien and Kerschensteiner, 2018). Calcium signaling has a pivotal role in this control through an interaction with calcium-binding proteins and sensors (Burgoyne and Haynes, 2015). The role of neuronal calcium sensor 1 (NCS-1) in regulating calcium-dependent exocytosis, long-term depression, axonal growth, calcium channel function, and synaptogenesis has been described (McFerran et al., 1998; Hui and Feng, 2008; Jo et al., 2008; Weiss et al., 2010; Dason et al., 2012). NCS-1 has several proteins identified as potential interaction partners that explain its different roles (Boeckel and Ehrlich, 2018), but it interacts with the guanine-exchange factor Ric8a, which is essential for controlling the number of synapses and the probability of the release of a neurotransmitter (Romero-Pozuelo et al., 2014).

We previously found that phenothiazine FD44 is an inhibitor of the NCS-1/Ric8a interaction by performing virtual screenings from a chemical library containing over a thousand of small heterocyclic compounds. Subsequently, structural studies revealed that FD44 stabilized a dynamic C-terminal helix inside the hydrophobic crevice of NCS-1, making the complete interaction groove unavailable for Ric8a binding. FD44 yielded a stable compound with a low toxicity in neuronal cultures. Unlike chlorpromazine, an antipsychotic drug with a similar structure, FD44 does not interact with neuronal receptors such as dopamine (DA) receptors (D1R and D2R). More importantly, FD44 showed an effect *in vivo* and restored synapse number and associative learning in a *Drosophila melanogaster* disease model of FXS (Mansilla et al., 2017).

In this work, we show that FD44 is a good inhibitor in cells with adequate oral bioavailability and brain penetration. FD44 has been demonstrated to rescue behavioral phenotypes in mice caused by FMRP losses such as hyperactivity, anxiety, stereotype, aggression, and social disorder. Dopaminergic neurons from the ventral tegmental area (VTA), which project to the prefrontal cortex and limbic system, are required to control these behaviors (Fields et al., 2007; Scott-Van Zeeland et al., 2010; Bariselli et al., 2018). Here, we show that limbic DA metabolism is increased in the FXS mouse model and that this neurotransmitter dysregulation can be reversed by FD44 treatment.

In summary, we have taken a step toward establishing the inhibition of the NCS-1/Ric8a complex as a treatment for FXS or other neurodevelopmental disorders associated with synaptic homeostasis dysfunction. We show that the small compound FD44 is an excellent therapeutic drug candidate for FXS, and we suggest that the metabolism of DA is a new biochemical marker for FXS.

## Materials and methods

### Cell culture and transfection

Human embryonic kidney HEK293 cells were grown in Dulbecco's modified Eagle's medium supplemented with 2 mM L-glutamine, 100 U/ml penicillin/streptomycin, MEM non-essential amino acid solution (1/100), and 5% (v/v), and heat-inactivated fetal bovine serum (all from Thermo). The human NCS-1 and Ric8a-V5 constructs were previously generated (Mansilla et al., 2017). Plasmids were co-transfected onto HEK293 using Lipofectamin 2000 (Thermo) following the manufacturer's instructions. Approximately 20 μM of FD44 diluted in dimethyl sulfoxide (DMSO) or the same volume of DMSO was added to culture cells 24 h after transfection.

### Proximity ligation assay

For the *in situ* proximity ligation assay (PLA), cells were grown in coverslips and, after transfection and treatment, fixed for 20 min with a fixation reagent, and later permeabilized with a permeabilization reagent, all from the Intracellular Fixation and Permeabilization buffer set (eBioscience, Thermo). This set allows a smooth fixation and an adequate permeabilization, indicated to carry out flow cytometry experiments. A mixture of the primary antibodies: mouse anti-V5 (1:2,000; Thermo) and rabbit anti-NCS-1 (1:1,000; cell signaling) diluted in the permeabilization reagent was used as a first step. Secondly, cells were incubated with the Duolink *in situ* PLA anti-rabbit MINUS and anti-mouse PLUS probes (Sigma), negative controls without primary antibodies are included in each experiment. Finally, interactions were detected with Duolink *in Situ* PLA Green Detection kit (Sigma) following the manufacturer's instructions. Cover slips were mounted with ProLong reagent (Thermo) containing Hoescht, and staining was analyzed with a standard fluorescence microscope.

To better quantify the interaction, PLA was performed followed by flow cytometry. Cells were detached, dissociated with trypsin and rapidly fixed, then washed, permeabilized, and incubated with primary antibodies as described above. The interaction was revealed by Duolink PLA flow cytometry green kit (Sigma) following the manufacturer's instructions. Cells were analyzed on FACS Canto II (BD Bioscience) with BD FACS Diva software.

### Animals

In this study, we have used two FXS mouse models, the Fmr1 KO and the Fmr1 KO2 and their corresponding wild-type (WT) littermates. Fmr1 KO and KO2 mice lack both protein and mRNA and recapitulate behavioral symptoms observed in human FXS pathology, including hyperactivity, repetitive behaviors, and deficits in learning and memory (Kazdoba et al., 2014; Gaudissard et al., 2017).

Fmr1 KO was supplied by The Jackson Laboratory (JAX stock #003025, strain B6.129P2-Fmr1tm1Cgr/J). Targeted mutation was performed in the laboratory of Dr. Ben Oostra at Erasmus University in the Netherlands by replacing exon 5 of the *fmr1* gene with a neomycin resistance cassette (Bakker et al., 1994). These mice were backcrossed to C57BL/6J for a total equivalent of nine generations.

Fragile X mental retardation 1 KO2 mice were provided by FRAXA Research Foundation, MA, USA. Fmr1 KO2 mice were generated by deleting the promoter and first exon of *fmr1* gene (Mientjes et al., 2006) and then were backcrossed to a C57BL/6J background for more than eight generations.

The pharmacokinetic (PK) assay was performed by Sai Life Sciences Ltd. in BALB/c WT mice aged 8–12-months.

Heterozygous breeding pairs were used to generate WT and KO littermates for all studies with FXS models. Mice were housed in four to five per cage groups of the same genotype in a temperature- (21 ± 1°C) and humidity-controlled room with a 12-h light–dark cycle. Food and water were available *ad libitum*.

### Animal treatment and PK profile

FD44 solution formulation in 5% NMP, 5% Solutol HS-15, 30% PEG-400, and 60% citric acid (10 mM) was administered in doses of 10 and 50 mg/kg dose intraperitoneally and orally, respectively. A single dose was administered to 8–12-month-old mice for PK and a daily intraperitoneal dose to 2-month-old mice for 4 weeks for further behavioral and biochemical studies. FD44 was well tolerated by Fmr1 KO, Fmr1 KO2, and WT mice, as any clinical signs were not observed. PK was performed in collaboration with Sai life Science Ltd. Blood samples were collected from retro-orbital plexus into ethylenediamine tetraacetic acid (EDTA) tubes from a set of three mice at each time point. Plasma was immediately harvested from the blood by centrifugation at 2,500 g for 10 min at 4°C. Immediately after collection of blood, brain samples were collected and homogenized using ice-cold phosphate buffer saline. Concentrations of FD44 in mouse plasma and brain samples were determined by the fit-for-purpose liquid chromatography with tandem mass spectrometry (LC-MS/MS) method. PK parameters were calculated using the non-compartmental analysis tool of Phoenix WinNonlin^®^ (Version 7.0). Areas under the concentration time curve (AUC_last_ and AUC_inf_) were calculated using a linear trapezoidal rule.

### Behavioral tests

Tests were conducted at the end of the treatment, that is, 4th week, and mice were tested on one behavioral task a day. The open field test was used to determine hyperactivity and habituation to a novel environment. For Fmr1 KO2 mice, open field was performed in a VersaMax activity monitor chamber (AccuScan Instruments). Activity was monitored by IR beam breaks and decoded using VersaDat software (AccuScan). For Fmr1 KO, an IR Actimeter (Panlab) was used to measure activity, followed by an analysis with ActTitrack software (Panlab). The distance traveled for 30 min was measured in centimeter for all cases.

Associative learning of an aversive sound followed by an electrical shock was used to analyze fear conditioning. For this, mice were given a 120-s habituation period in the exploratory chamber before the first two identical trials (210 s apart) to allow exploration of the chamber. An 80-dB auditory cue was then presented (15–30 s) with a mild foot shock (0.6 mA, 1 s) administered during the last 2 s of the tone presentation, which co-terminated with the tone. Memory was tested 24 h after training, and freezing time was recorded for 5 min.

Stereotype was evaluated by self-grooming. For this, mice were placed individually in a cage, after a 5-min habituation period, the time spent to grooming any part of the body was recorded for 3 min. For the aggression test, experimental “test” and WT “control” mice were placed in the testing cage simultaneously. Tail rattling, bites, or mounts were considered as an attack. The latency between attacks was measured for 3 min.

The three-chamber test assesses cognition in the form of interest in social novelty. Testing occurs in a three-chambered box with openings between the chambers. After habituating to the empty box, the test mouse encounters a never-before-met intruder (unfamiliar) under one pencil cup in one chamber and a second familiar mouse (contact for 6 h previous to the test) under another pencil cup in the opposite chamber. The time spent in each chamber is quantified for 6 min.

### DA levels and DA metabolic profile

Mice were sacrificed by cervical dislocation followed by rapid decapitation; brain regions were immediately dissected on an ice-cold glass plate.

Tissue samples were preserved at −80°C until analysis. The limbic and prefrontal cortex samples were sonicated in 6 vol. (weight/volume) of 0.4 N perchloric acid for deproteinization and centrifuged at 10,000 g at 4°C for 20 min, and then the supernatant was used for high-performance liquid chromatography (HPLC) analysis. DA and its metabolites were measured from supernatants by HPLC with an ESA Coulochem detector, according to Mena et al. (1993). Chromatographic conditions were as follows: an ACE 5 C18, 150 × 4.6 mm (UK) column, equilibrated in citrate/acetate buffer 0.1 M, pH 3.9 with 10% methanol, 1 mM EDTA, and 1.2 mM heptane sulfonic acid. Monoamine levels were identified by their retention time, and amounts were calculated against calibrated external standard solutions. Data are referred to as percentage of the baseline condition WT-V.

### RNA extraction and quantitative Reverse transcription polymerase chain reaction (qRT-PCR)

Ribonucleic acid extraction from frozen dissected brain regions was performed using Trizol (Thermo). Subsequently, retro transcription with the Superscript III kit (Fisher) and quantitative polymerase chain reaction (qPCR) analysis using Taqman specific probes (Applied Biosystems) for NCS-1, Ric8a, D1R, D2R, and the glyceraldehyde 3-phosphate dehydrogenase (GAPDH) gene that was amplified as the housekeeping gene to control RNA input. The PCR reaction was performed with Taqman Fast Universal Master Mix (Applied Biosystems). The relative quantification method with standard curves was used, and the samples were normalized for GAPDH mRNA levels. Data are represented as fold change to the basal (WT-V) condition. The qPCRs were carried out in StepOne Plus qPCR (Applied Biosystems).

### Statistical analysis

Results were expressed as mean ± standard deviation (SD). Statistical analysis and graph plotting were performed with the GraphPad Prism software. In an experiment with only two experimental groups, a Student's *t*-test is performed. A one way ANOVA analysis followed by multiple comparisons Bonferroni or Sidak's test was used in assays with more than two groups. Significant differences were considered when *p* ≤ 0.05.

## Results

### FD44 inhibition of NCS-1/Ric8a complex *in situ*

Among compounds previously identified as inhibitors of the NCS-1/Ric8a complex, compound FD44 had the best solubility profile, low neutron toxicity, and an almost total inhibitory effect in a binding assay *in vitro* (Mansilla et al., 2017). The *in vitro* binding assay is based on a co-immunoprecipitation of NCS-1 with Ric8a from a cell lysate and, although it has been useful to screen potential protein–protein interaction modulators, this assay does not reproduce the intracellular physiological conditions where the interaction take place.

**Figure 1:**
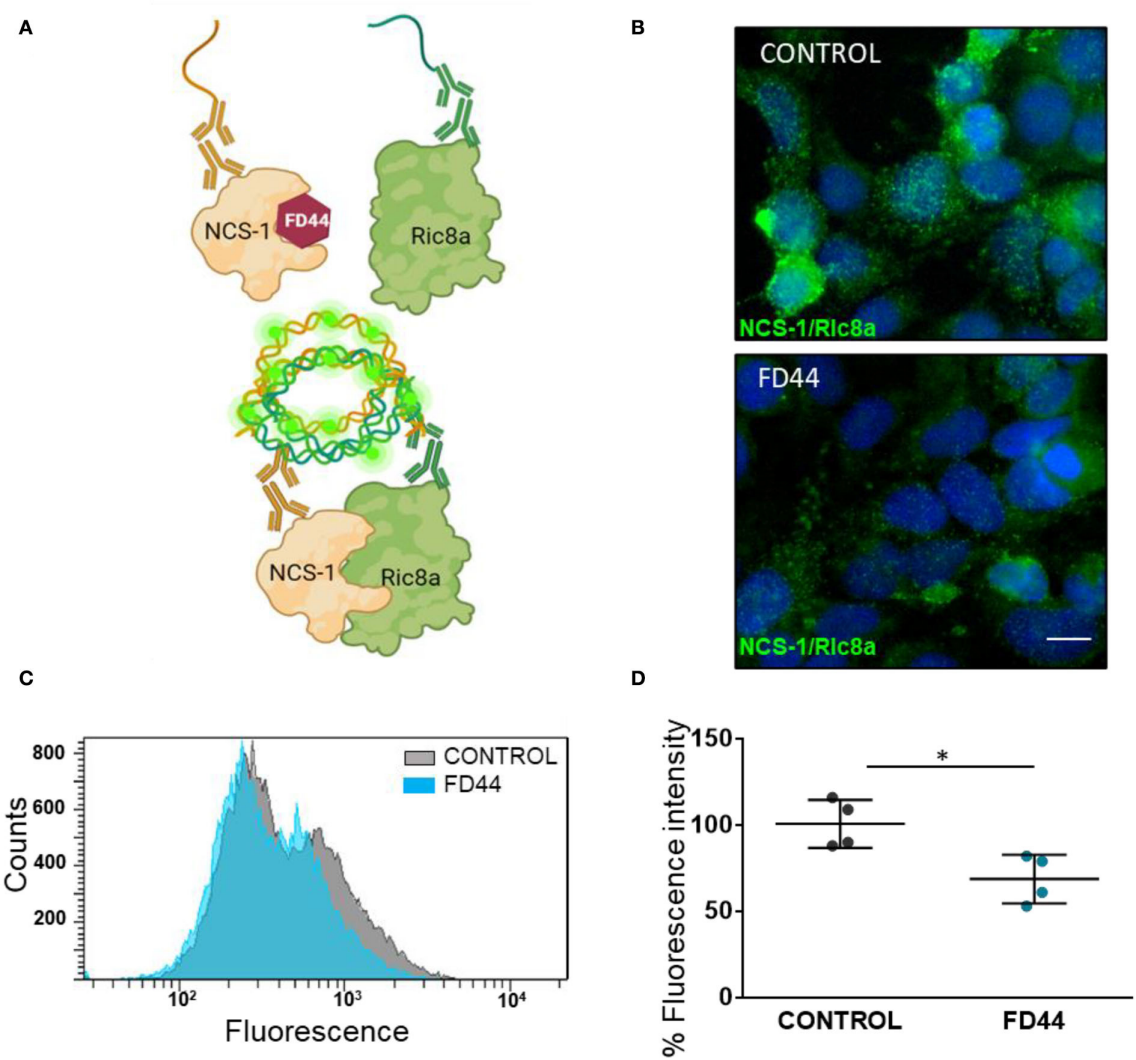
*In situ* analysis of neuronal calcium sensor 1 (NCS-1) binding to Ric8a. **(A)** Schematic representation of the basis of a proximity ligation assay (PLA) for the study of the NCS-1/Ric8a interaction. FD44 blocks the binding of Ric8a by making the NCS-1 interaction crevice unavailable for Ric8a. When the complex is not formed, probes of the PLA secondary antibodies do not bind, and no fluorescence is detected. In contrast when FD44 is not present, the complex is formed, the probes ligate and subsequently elongate, and fluorescence is detected. **(B)** PLA was performed in HEK293 co-transfected with NCS-1 and Ric8a-V5 and treated the last 24 h with vehicle (V; control) or FD44 (20 μM). After fixation, PLA was performed and a green fluorescence probe was used to reveal the binding. Nuclei are labeled with Hoechst. Scale bar corresponds to 10 μm. **(C)** PLA was performed as in B, but in this case cells were analyzed by flow cytometry. The histogram showing the number of cells (counts) per green fluorescence of a representative experiment is shown. **(D)** Graph showing the mean fluorescence intensity of treated HEK293 overexpressing NCS-1 and Ric8a-V5 following fixation and PLA for flow cytometry. Graph represents 15,000 cells in four independent experiments. Values are expressed as mean ± standard deviation (SD) *n* = 4. Statistical analysis performed by Student's *t*-test. ^*^p < 0.05, NCS-1/Ric8a treated with solvent (CONTROL), NCS-1/Ric8a treated with FD44 (FD44).

To study the inhibition of NCS-1/Ric8a binding in more physiological conditions, we have used the Duolink PLA technology, which allows the evaluation of protein–protein interactions *in situ* with high sensitivity (Alam, 2022). This tool is based on the recognition of specific antibodies of the two proteins of interest, NCS-1 and Ric8a. It makes use of deoxyribonucleic acid (DNA) primers that are covalently linked to secondary antibodies that, after hybridization, allows PCR amplification with fluorescent probes to be later visualized as individual fluorescence spots using microscopy or flow cytometry (Figure 1A).

HEK293 cells were co-transfected with human NCS-1 and Ric8a-V5 expressing constructs and treated with FD44 or the same amount of vehicle DMSO as control. After PLA, the intensity and number of green fluorescence spots representing the NCS-1/Ric8a binding are reduced in FD44-treated cells (Figure 1B). To better quantify inhibition, we carried out PLA followed by the flow cytometry analysis. Uniparameter histograms showing the number of cells per fluorescence intensity, show a displaced and smaller curve in FD44-treated cells (Figure 1C). A 40% reduction in binding in FD44-treated cells was calculated after quantification of four independent flow cytometry experiments (Figure 1D).

### FD44 PK profile

Pharmacokinetics provides new insights into the metabolism and bioavailability of drugs and is one of the most important techniques for calculating effective doses needed for further mice treatment, as well as for explaining and predicting efficacy-related events. In the case of FD44, we also needed to study how it penetrates the brain, since it is intended to treat brain diseases. This led us to carry out a complete study of the kinetics of the compound in two routes: intraperitoneal and oral.

**Figure 2:**
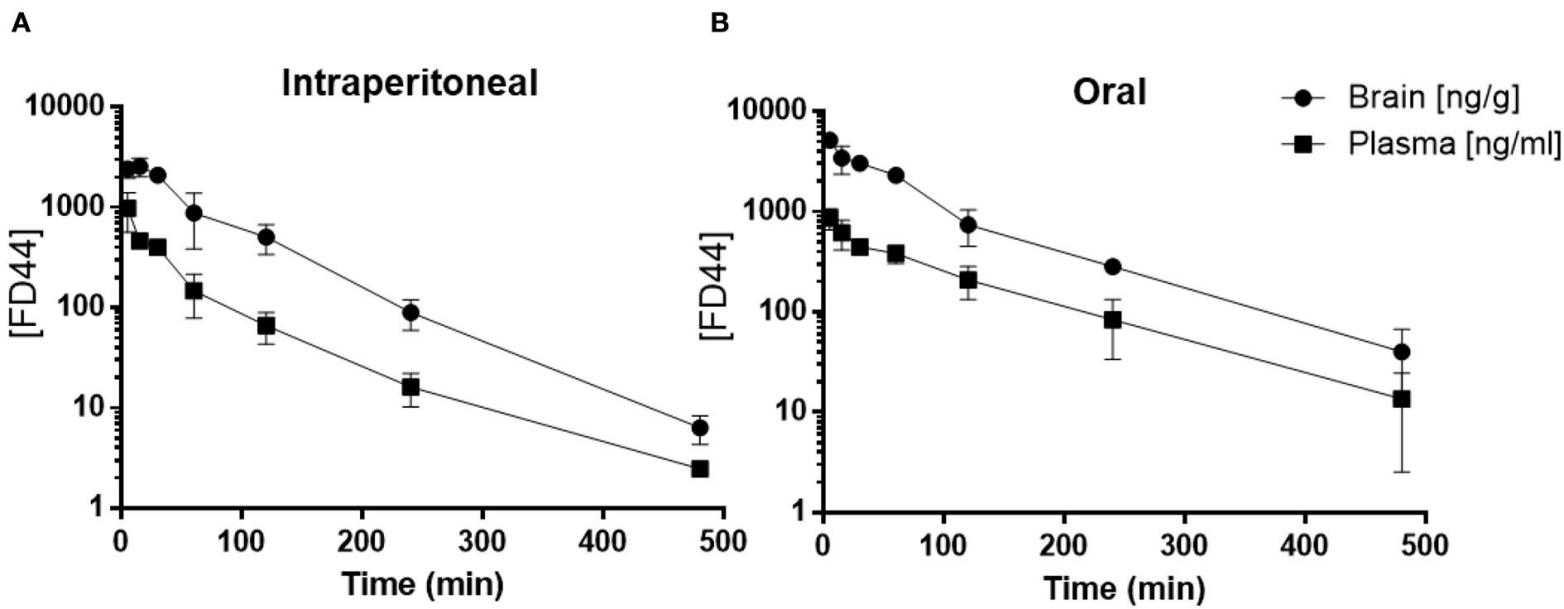
FD44 pharmacokinetic (PK) profile. **(A)** Mean ± SD of plasma (square) and brain (circle) FD44 concentration over time (5, 15, 30, 60, 120, 240, and 480 min), following FD44 single intraperitoneal administration (dose: 10 mg/kg) or **(B)** oral administration (dose: 50 mg/kg). The concentration of FD44 [FD44] is presented in a semilogarithmic graph, units in plasma are ng/ml and in brain ng/gr. *n* = 3 mice per time point.

Following a single intraperitoneal administration of FD44 at 10 mg/kg dose, plasma concentrations were quantifiable up to 480 min. The time to reach maximum concentration (*T*_max_) is 8 min. Brain concentrations were quantifiable up to 480 min, and *T*_max_ is 25 min. Brain-to-plasma ratio for the intraperitoneal route ranged 2.48–7.65. (Figure 2A; Table 1)

**Table 1:** Pharmacokinetic (PK) parameters for FD44 summary.

**Route**	**Dose** **(mg/kg)**	**Matrix**	**C_max_** **(ng/mL)**	**T_max_** **(min)**	**AUC_last_** (min*ng/mL)	**AUC_inf_** (min*ng/mL)
IP	10	Plasma	981.19	5	595.59	617.38
		Brain	2560.16	15	3333.30	3341.58
PO	50	Plasma	858.90	15	1947.35	1970.42
		Brain	5164.38	15	10379.95	10443.24

After intraperitoneal (IP) or oral (PO) administration of FD44 in a single dose, maximum concentration (C_max_) and the time to reach C_max_ (T_max_) is calculated. The area under the concentration (AUC) time curve reflects the total amount of drug that has effectively reached the target (blood or brain) along time and depends on the degree of bioavailability and the rate of elimination from the body. The AUC during the period of observation (AUC_last_) and the AUC from zero to infinity (AUC_inf_) are shown for each administration route.

Oral administration of FD44 at a single dose of 50 mg/kg yielded quantifiable plasma and brain concentrations up to 480 min, and *T*_max_ was 25 min. The brain-to-plasma ratio ranged from 2.90 to 6.81 (Figure 2B; Table 1).

This study reveals that FD44 perfectly crosses the blood–brain barrier and availability up to 480 min and allows us to propose a treatment in mice with a daily intraperitoneal dose of 10 mg/kg.

### Behavior phenotypes in two FXS mouse models treated with FD44

We have previously shown that administration of FD44 in the food of *D. melanogaster* FMRP mutants or in a *fmr1* RNAi strain, restored synapse number and associative learning (Mansilla et al., 2017). To determine if the FD44 effect extends to a vertebrate model, we have treated two FXS mouse models.

Behavioral tests in FXS mouse models have yielded different and sometimes conflicting results. It is important to consider the experimental design, but also the mouse model and even the genetic background within the same model (Pietropaolo et al., 2011). For this reason, in this study, we used two mouse models, which allows us a broader and more robust approach.

**Figure 3:**
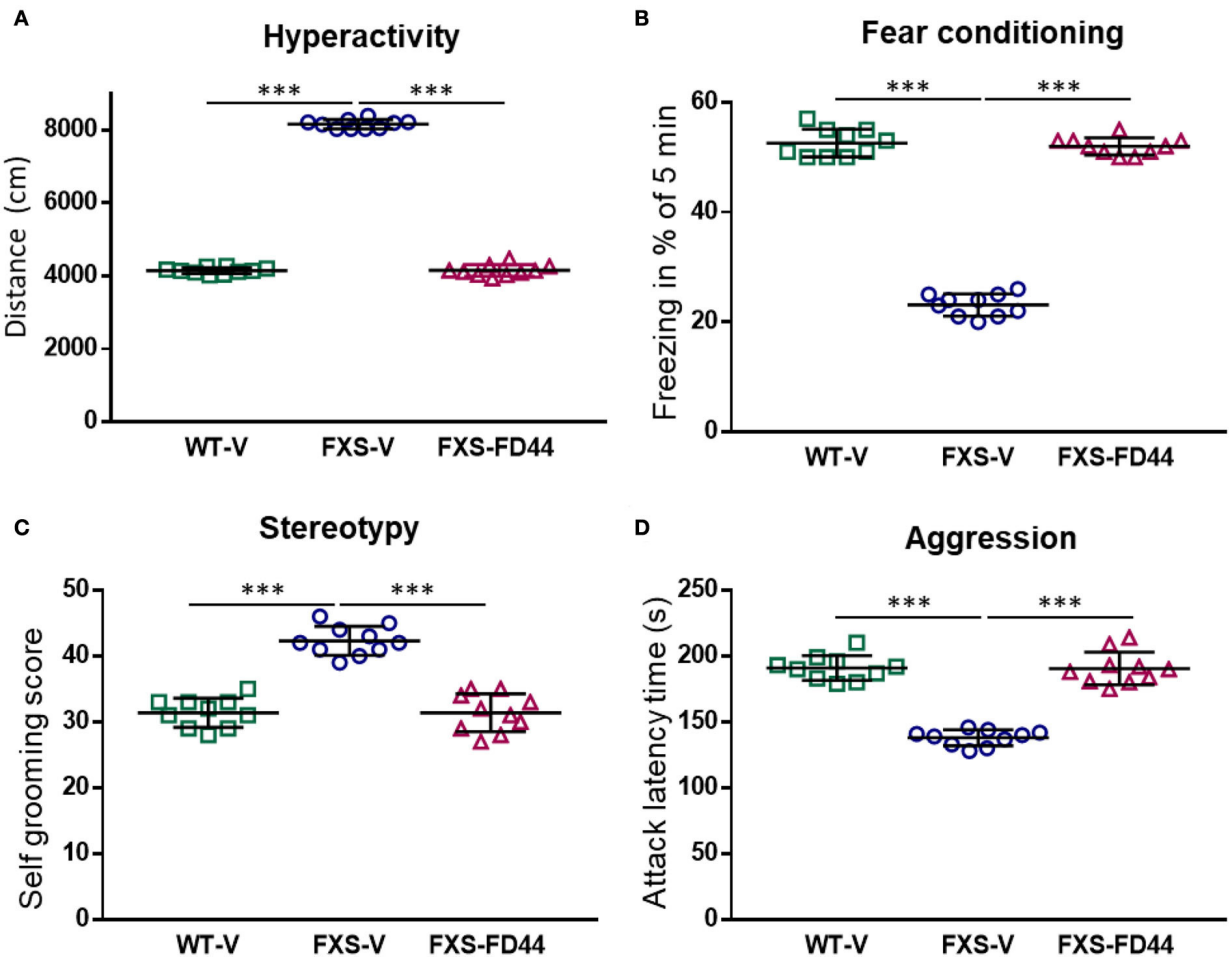
FD44 effects on the behavior in fragile X mental retardation 1 (Fmr1) KO2 mice. Wild-type (WT) or Fmr1 KO2 [Fragile X syndrome (FXS)] adult mice were treated intraperitoneally with V or with 10 mg/kg of FD44, daily for 4 weeks and then subjected to a range of behavioral tests. FD44 treatment caused a significant improvement of Fmr1 KO2 phenotypes in **(A)** hyperactivity, measured by the distance traveled in the open field, **(B)** associative learning, measured by the freezing response after fear conditioning with an aversive stimulus, **(C)** stereotype revealed by self-grooming, and **(D)** aggression, measured by the latency between attacks. Data were analyzed by one-way ANOVA followed by Sidak's corrected multiple pair-wise comparison. *n* = 10 mice per group, ^***^p < 0.001.

First, we performed FD44 treatment on the FXS model Fmr1 KO2. Behavioral studies were carried out after 4 weeks of treatment, and a range of behavioral studies involving different brain circuits were completed. The open field has been widely used for hyperactivity assessment. Fmr1 KO2 mice showed approximately twice the level of activity as WT littermates in the open field test, showing a significant difference between vehicle-treated groups and complete recovery with FD44 treatment (Figure 3A).

Working memory and hippocampal-independent recognition memory are normal in FXS mice, while memory recall after training has been shown to be altered (Li et al., 2020). To evaluate contextual memory formation and recall, we have performed the fear conditioning test, which assesses the animal's ability to make an association between an environmental cue (sound) and a paired foot shock, which results in quantifiable freezing. The results here showed a significant difference between the WT-V and FXS-V groups in freezing behavior and FD44 treatment completely rescued impaired associative learning (Figure 3B).

Other behaviors reminiscent of patients with FXS are perseveration and repetitive behaviors (Reisinger et al., 2020). To test the hypothesis of whether NCS-1/Ric8a complex inhibition can rescue such alterations, self-grooming was analyzed in Fmr1 KO2-treated animals. A high level of stereotype in vehicle-treated FXS mice was restored by FD44 treatment (Figure 3C).

A large proportion of individuals with FXS show irritability and aggression (Britton et al., 2020). These aberrant behaviors can be easily evaluated in mice by measuring aggressive behavior (mounting, biting, or chasing) toward a conspecific. To test the hypothesis that FD44 could modify aggressiveness, the latency between attacks in Fmr1 KO2 was measured. FD44-treated FXS mice showed the low level of aggressiveness similar to their WT counterparts (Figure 3D).

As in other syndromic forms of autism, social behavior deficits are common in FXS (Cregenzán-Royo et al., 2022). Among all the tests available to study socialization in mice, the one that evaluates the greatest number of aspects of socialization, including recognition, motivation, and memory, is the one in which the mouse is confronted with a familiar or an unknown conspecific. This type of test cannot be performed when the level of aggressiveness of the animal is very high, since the interest in socializing with one or another animal remains in the background, compared to a more primary aggressive behavior.

**Figure 4:**
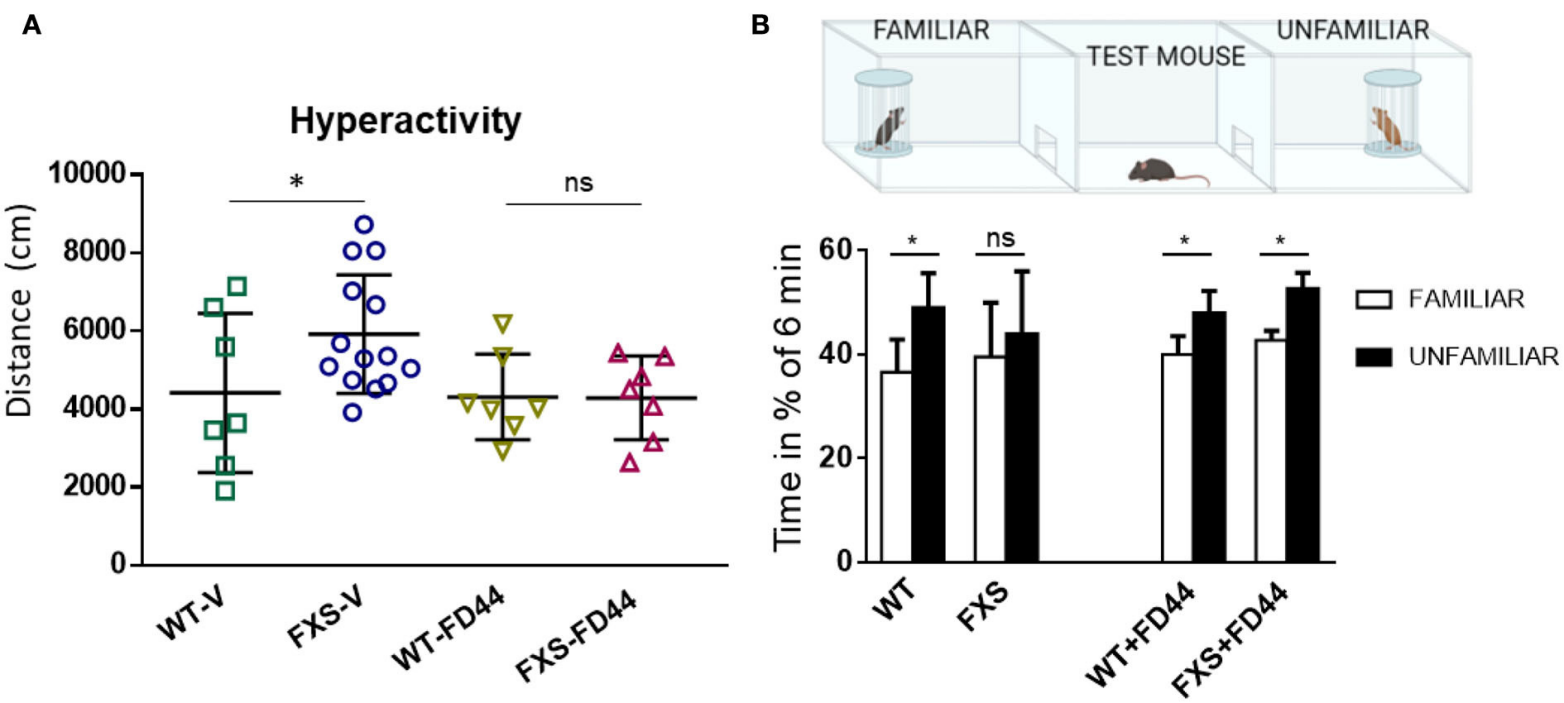
FD44 effects on hyperactivity and social approach in Fmr1 KO mice. WT or Fmr1 KO (FXS) adult mice were treated intraperitoneally for 4 weeks, with V or with 10 mg/kg FD44. **(A)** An open field test was performed. Hyperactivity, measured as the traveled distance, was significantly higher in the V-treated FXS compared to WT. **(B)** A three-chamber social test was performed, as indicated in the picture. Bars represent the mean percentage of time the test mice in each group spent in the right chamber with the familiar mouse (white column) vs. the percentage of time in the left chamber with the unfamiliar mouse (black column). Untreated FXS mice showed a nonsignificant (ns) preference. Data were analyzed by one-way ANOVA followed by Bonferroni multiple pair-wise comparison. *n* = 7 (WT-V), 14 (FXS-V), 7 (WT-FD44), and 7 (FXS-FD44), only relevant comparisons are shown, ^*^p < 0.05 and ns = nonsignificant.

To test the effect of FD44 on socialization, we have used the Fmr1 KO animal, whose level of aggressiveness is low, just like WT (Mineur et al., 2006 and our personal observation), and in contrast to the high level of aggression behavior observed for the Fmr1 KO2 (Figure 3D). The Fmr1 KO do exhibit behavioral phenotypes typical of FXS, but have been found to be milder. For example, as we show here, in terms of hyperactivity, measured as distance traveled in the open field, there is a slight but significant increase in activity in FXS-V, which normalizes after treatment with FD44 (Figure 4A). To study socialization in these mice, we have used the three-chamber assay in which a test mouse has to choose between spending time in the chamber with a familiar mouse (previous 6 h contact) or with a totally unknown mouse (Figure 4B). Under the untreated FXS condition (FXS-V), social behavior deficit is shown as a nonsignificant preference for any mouse, in contrast to the condition of WT-V-, WT-FD44-, and FXS-FD44-treated mice, in which animals spent significantly more time exploring the chamber with the unfamiliar mice, as a natural interest in the unknown (Figure 4B). Therefore, FD44 treatment was able to restore the impaired social interest.

### DA metabolism in FXS

Defects in the DA signaling pathways have been linked to FXS (Wang et al., 2008; Weinshenker and Warren, 2008; Paul et al., 2013), and the characteristic behavioral phenotypes of FXS are regulated by dopaminergic neurons from the VTA, whose projections modulate the activity of the limbic system and the prefrontal cortex (Fields et al., 2007; Bariselli et al., 2018). The molecular effects of DA are terminated by reuptake into presynaptic terminals or by conversion to main inactive metabolites: 3,4-dihydroxyphenylacetic acid (DOPAC) and homovanillic acid (HVA).

**Figure 5:**
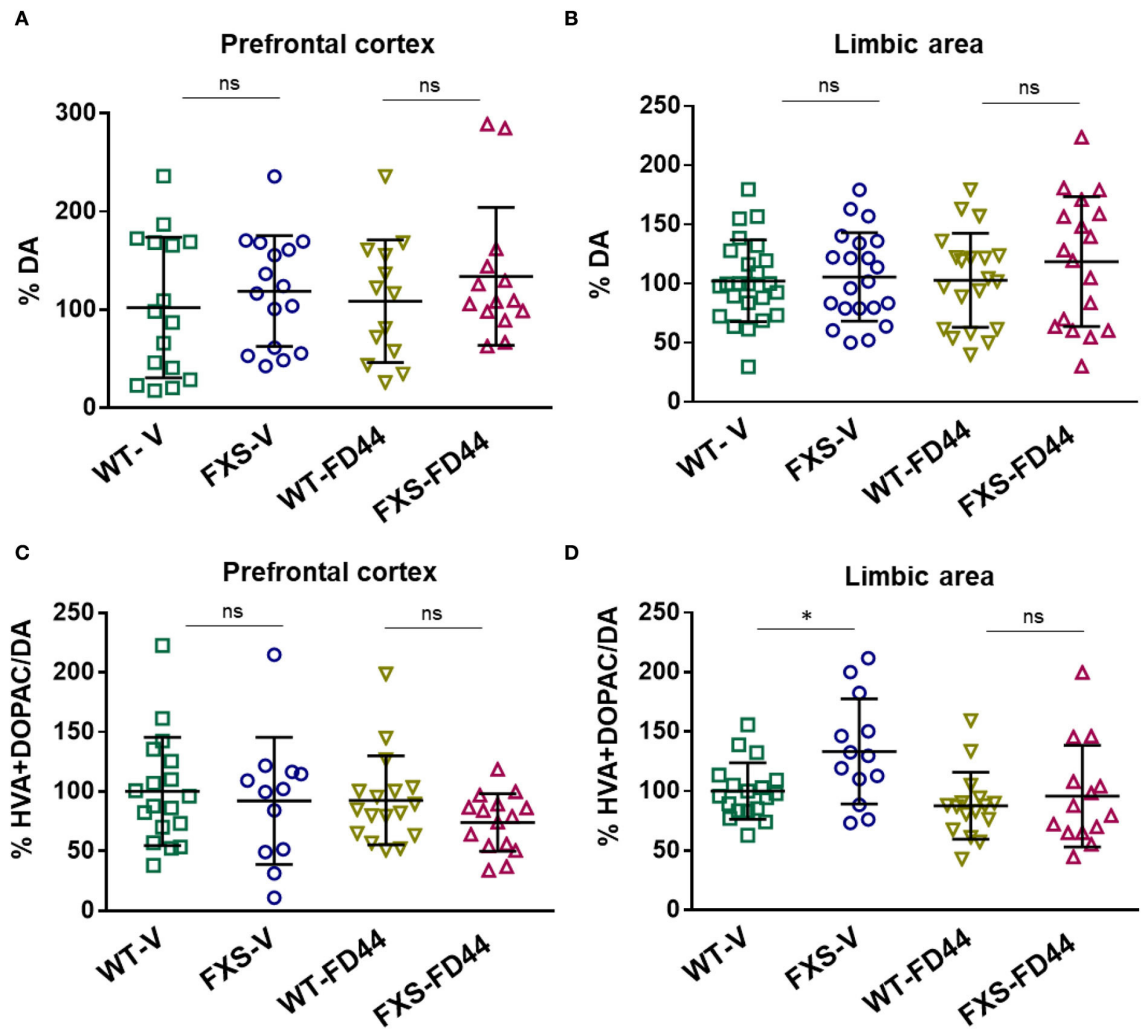
Dopamine (DA) levels and DA metabolic rate in the brain areas of Fmr1 KO mice. The prefrontal cortex and limbic area were dissected from WT or Fmr1 KO (FXS) adult mice after treatment with V or with FD44 intraperitoneally for 4 weeks. The percentage of DA referred to WT-V as baseline condition in the **(A)** prefrontal cortex and **(B)** the limbic system. DA metabolism was shown as the percentage of 3,4-dihydroxyphenylacetic acid (DOPAC) and homovanillic acid (HVA) vs. DA in the **(C)** prefrontal cortex and **(D)** limbic system. Data were analyzed by one-way ANOVA followed by Bonferroni multiple pairwise comparison. *n* = 12-21. Each FXS group is compared to its WT counterpart, other comparisons were not relevant ^*^p < 0.05, ns, nonsignificant.

To evaluate whether DA metabolism is associated with behavioral phenotypes in Fmr1 KO mice, we have measured the amount of DA and main metabolites (HVA + DOPAC), in the limbic area and the prefrontal cortex. We found no differences in the amount of DA, but the DA metabolism ratio (metabolites/DA) was significantly increased in the limbic area while the prefrontal cortex remained unaffected (Figure 5). As shown for other phenotypes, this biochemical difference was reversed when mice were treated with FD44 (Figure 5D).

### NCS-1, Ric8a and DA receptors expression levels

**Figure 6:**
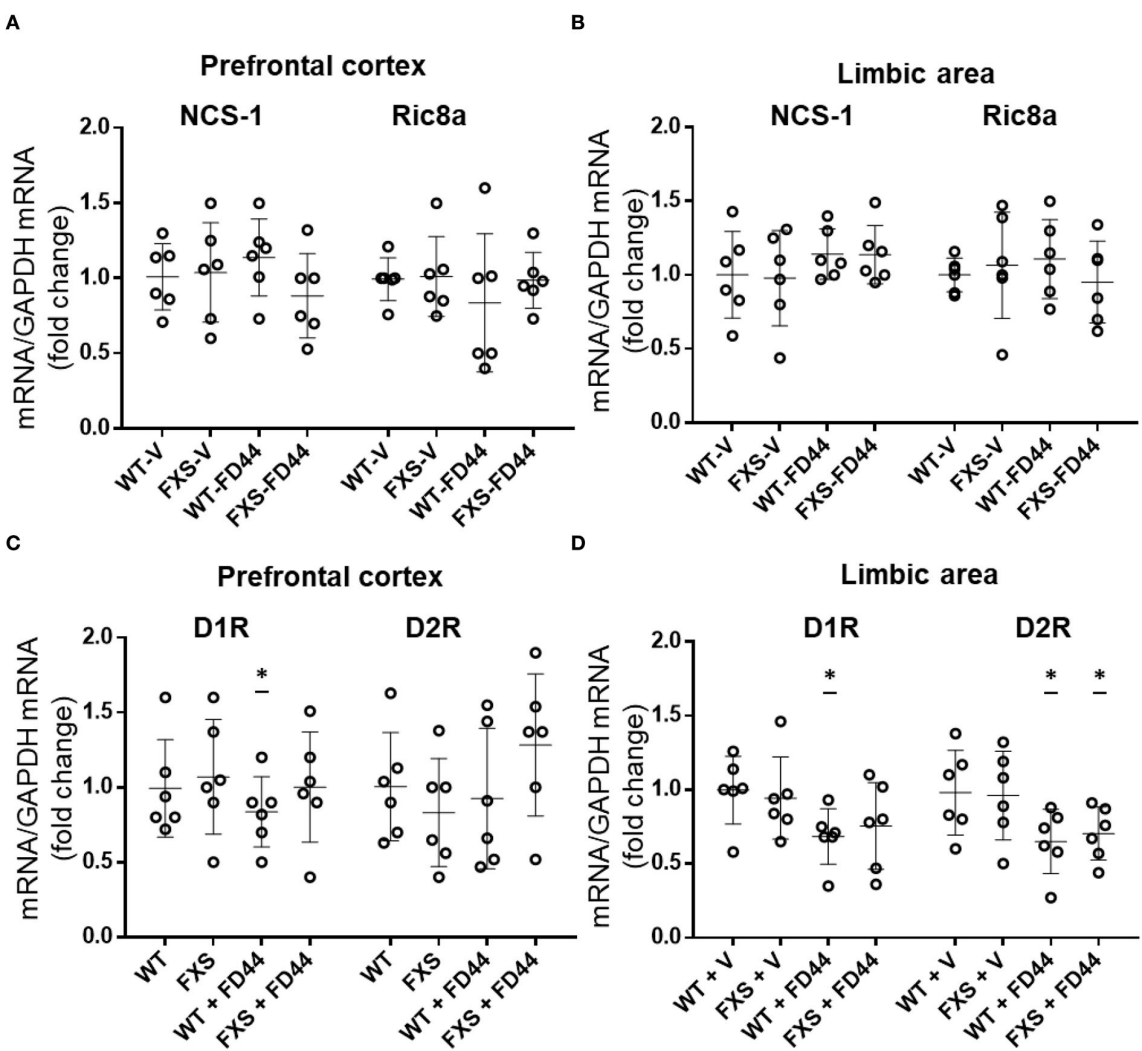
Expression profile of NCS-1, Ric8a, and DA receptors D1 and D2. Ribonucleic acid (RNA) from the limbic system and prefrontal cortex of Fmr1 KO (FXS) or WT mice was extracted after treatment with V or with FD44 intraperitoneally for 4 weeks, and qRT-PCR was performed. **(A)** The messenger RNA (mRNA) levels of NCS-1 and Ric8a normalized with glyceraldehyde 3-phosphate dehydrogenase (GAPDH) mRNA are shown in the prefrontal cortex or **(B)** limbic area. **(C)** The mRNA levels of DA receptor D1R and D2R normalized with GAPDH mRNA are shown in the prefrontal cortex or **(D)** limbic area. Data were analyzed by one-way ANOVA followed by Bonferroni multiple pair-wise comparison. Scatter plots show mean ± SD of six mice per condition measured in triplicates. Comparisons are performed with WT-V because it is considered the basal expression condition, and the fold change is represented in the graph. ^*^p < 0.05 is considered significant; otherwise it is nonsignificant.

We have shown that the compound FD44 is a protein–protein interaction inhibitor of NCS-1 and Ric8a (Figure 1; Mansilla et al., 2017), but this does not imply changes in the relative expression of the genes involved in the interaction. To test this hypothesis, qRT-PCR was performed to evaluate gene expression levels of NCS-1 and Ric8a from previously studied areas (the limbic system and prefrontal cortex). As shown in Figure 6A, no significant differences were found in either case.

As the DA receptor D2R has been identified as a binding partner of NCS-1 (Kabbani et al., 2002) and DA D1R is affected by the loss of function of FMRP (Wang et al., 2008), we thought it was relevant to include D1R and D2R in the gene expression profile and study the mRNA level of this gene in relation to FXS pathology and FD44 treatment. Surprisingly, there is a slight but significant decrease of D1R in WT-FD44 treated mice in both brain areas. In the limbic area, the D2R mRNA levels are decreased in FD44-treated mice (Figure 6B). We found only 0.2- and 0.4-fold changes in D1R and D2R gene expression, indicating that these changes were probably not physiologically relevant to each other (Figures 6C,D).

## Discussion

The possibility of finely regulating a cell process by inhibiting protein interactions, rather than totally blocking the activity of specific proteins, has been a success in the field of drug development (Arkin et al., 2014). The identification of “hot spots” in the NCS-1 interfacial area for Ric8a binding allowed us to find a small molecule, phenothiazine FD44, with a good drug profile and *in vitro* inhibition efficacy (Mansilla et al., 2017). Here, we have demonstrated the inhibitory efficiency of cells, their ability to cross the blood–brain barrier and, their role as disease modifiers in two FXS mouse models.

The inhibitory efficacy of the FD44 compound had been demonstrated in a previous work in cell lysates (Mansilla et al., 2017). However, we went a step forward and used a PLA to show for the first time that inhibition occurs in intact cells. This sensitive technique proved that FD44 inhibits the protein–protein interaction in the natural cellular context of the cell membrane, where NCS-1 is constantly bound, proving its activity *in vivo*. Moreover, 40% inhibition was still detected after long exposure with FD44, which indicates a high degree of efficacy.

The PK profile of FD44 indicates that this compound can cross the blood–brain barrier and reach the brain. In fact, it has a similar profile to the best known phenothiazines and the widely antipsychotic chlorpromazine (Shibanoki et al., 1984). These promising previous data justify the study presented here in a FXS mouse model.

Conflicting results have been reported regarding spinal density (Pfeiffer and Huber, 2009) or behavioral phenotypes in Fmr1 knockout mice (Kat et al., 2022). This is not surprising due to the experimental variability between laboratories, the difference in encounters between the genetic models and even the mouse background, which has turned out to be decisive. Fmr1 KO mice have been generated on C57BL/6 (B6) and FVB backgrounds. Magnetic resonance imaging (MRI) studies have observed multiple differences in the neuroanatomy of male Fmr1 KO mice on a FVB but not in the B6 background (Paradee et al., 1999; Lai et al., 2016). In fact, in monogenic diseases like FXS, genetic and environmental factors have a great influence, and these do not translate in all patients with FXS displaying autistic behaviors and intellectual deficits ranging from mild learning disfunction to severe cognitive impairment (Payán-Gómez et al., 2021). This variation should be a lesson rather than a problem, since it can partially explain why it has been impossible to transfer successful animal preclinical studies into clinical practice (Dahlhaus, 2018). Here, we have taken advantage of two mouse models. On one hand, Fmr1 KO2 mimics aggressive behavior and consequently presents difficulties in assessing behavioral tests on social preferences. On the other hand, the Fmr1 KO model has a low level of aggressiveness, which allowed us to perform the social preference task. Thus, hyperactivity, fear conditioning, repetitive behaviors, aggressiveness, and social preference could be evaluated, all of them were dependent on different brain circuits, and all of them recovered completely when treated with the compound FD44.

The prefrontal cortex is critical for many aspects of working memory, planning, and attention, and the limbic system is involved in a variety of fundamental cognitive and emotional tasks. The functionality of these areas is evident in specific behavioral tests, such as FXS mice presented here, which corresponds quite well with the symptomatology of the patient (Servadio et al., 2015). These brain areas of interest in FXS are regulated by midbrain nuclei, such as the VTA. A group of dopaminergic neurons whose diffuse projections of the so-called mesocortical and mesolimbic circuits modulate the activity of the prefrontal cortex and the limbic system, respectively (Douma and de Kloet, 2020). Therefore, VTA circuits are important in forming emotional–motivational valuations (Lloyd and Dayan, 2016) and DA signaling has been postulated as a main actor in the symptomatology of neurodevelopmental disorders (Cai et al., 2021). Specifically, a proteomic approach in *Drosophila fmr1* mutants indicated elevated DA synthesis (Zhang et al., 2005). We detected significant differences in DA levels, but instead we found a significant increase in DA metabolism in the limbic area of male Fmr1 KO mice compared to the limbic area of WT littermates. This is consistent with a previous study, which showed that female Fmr1 KO mice displayed increased DA turnover in cortical regions, the striatum, and the hippocampus, whereas KO males did not (Gruss and Braun, 2004). We were able to detect the difference in males probably due to the more sensitive technique used here.

Upon the release of DA into the synaptic cleft to interact with the postsynaptic DA receptors or the presynaptic DA autoreceptors, DA must be removed. It can either be recycled after the reuptake promoted by the DA transporter (DAT) or it can be degraded. The relative increase of HVA and DOPAC metabolites observed here can be explained by a lower reuptake rate or by an increase in the activity of DA degradation enzymes or both. In any case, an increase in the DA turnover and its associated phenotypes disappeared when FXS mice were treated with FD44.

Reduced striatal expression of DAT has been described in male Fmr1 KO mice (Chao et al., 2020). It is known that in pathological conditions such as Parkinson's disease, a decrease in DAT and a subsequent decrease in DA reuptake are connected to an increase in DA turnover (Sossi et al., 2007). In addition, increased DA turnover results in changes in the cellular redox state. Biochemical markers of oxidative stress have been detected in Fmr1 KO mouse brain (El Bekay et al., 2007). Increased DA metabolism may result in higher oscillations in synaptic DA concentration, which will explain some of the behavioral phenotypes found in FXS. Due to the direct connection of the DA metabolism phenotype with FXS symptomatology, we propose to use it as a biochemical marker for future studies.

Regarding DA receptors in FXS, DA receptor D1R signaling has been observed to be impaired in the forebrain due to subcellular redistribution of G protein-coupled receptor kinase 2 (GRK2) and subsequent phosphorylation of D1R (Wang et al., 2008). In our experiments, we have detected a small decrease in the expression of D1R and D2R genes, in some of the experimental groups always corresponding with FD44-treated mice. These minor differences are probably not physiologically relevant, and we cannot rule out whether active DA receptor levels are affected, which will be a more adequate way to evaluate dopaminergic signaling.

It is interesting to note that D2R and GRK2 have also been identified as NCS-1 binding partners (Kabbani et al., 2002). This is not surprising since NCS-1 is a central molecule in the regulation of synaptic homeostasis (Bandura and Feng, 2019). Although the binding mechanisms with partners other than Ric8a are not fully understood, the available structural data on various NCS-1/target complexes suggest that different mechanisms operate for each recognition process, in which calcium levels play an important role (Burgoyne and Haynes, 2015; Mansilla et al., 2017). In this line of evidence, FD44 could target a specific protein–protein interaction, although it is quite possible that inhibition of the Ric8a interaction increases the chance that free NCS-1 will interact with other binding partners. Therefore, our therapeutic approach for FXS is a small change involving the NCS-1 interaction network, where NCS-1 activity or levels are not compromised. In *D. melanogaster*, the simultaneous overexpression of NCS-1 and Ric8a suppresses the synaptic phenotypes found for each separate genetic overexpression and yields a normal number of synapses, just as when *fmr1* mutant flies are treated with FD44 (Romero-Pozuelo et al., 2014; Mansilla et al., 2017). We speculate that the inhibition of NCS-1/Ric8a interaction by FD44 can be equivalent to a simultaneous excess of free NCS-1 and Ric8a.

Taken together, we can conclude that the inhibition of the NCS-1/Ric8a complex with compound FD44 is capable of recovering all the biochemical and behavioral phenotypes found in the two FXS mouse models.

The activity of NCS-1 as a calcium sensor makes it a very interesting therapeutical target since most of the relevant pathways in brain function and development require calcium signal transduction through sensors. The regulation of the activity of these sensors can be the key to interfere with these pathways. In this work, we have proposed a novel therapeutic approach for FXS, not aimed at correcting the expression of a particular gene, but at modulating the protein interaction profile of a master regulator, that is, NCS-1.

## Data availability statement

The raw data supporting the conclusions of this article will be made available by the authors, without undue reservation.

## Ethics statement

Procedures with Fmr1 KO animals were in accordance with Spanish legislation (RD 53/2013) and the European Union Council Directive (2010/63/EU). The Ethics Committee of the Hospital Ramón y Cajal and the competent authority in Comunidad de Madrid approved all the protocols related to these animals. Experiments with Fmr1 KO2 were conducted in line with the requirements of the UK Animals (Scientific Procedures) Act, 1986. All procedures for animal maintenance and experimentation were approved and followed the recommendations of the Ethics Committee of the Institute of Ecology and Biodiversity (IEB), Faculty of Sciences of the University of Chile, and complied with Chilean regulations. PK assay was performed on BALB/c mice, procedures were performed in accordance with the guidelines provided by the Committee for the Purpose of Control and Supervision of Experiments on Animals (CPCSEA) as published in The Gazette of India, 15 December 1998, and approval from the Institutional Animal Ethics Committee was obtained before the initiation of this study.

## Author contributions

PC performed the experiments with the Fmr1 KO 2 mice. LF-B performed the experiments with the Fmr1 KO mice. MC performed the HPLC measures of catecholamines. SS-Y makes the PLAs and the qRT-PCR experiments. ER-M contributed with the flow cytometry analysis. MS-B contributed with the data analysis, interpretation, and critical revision of the manuscript. AG-R, CG, and AnM produced the FD44 compound and analyzed the PK experiments. AlM contributed with the conception, design of the study, data analysis, interpretation, and drafting the manuscript. All authors contributed to the article and approved the submitted version.

## Funding

This work was supported by the Caixa Research foundation (Caixa Impulse program CI18-00026) from Spain, the Fragile X Research Foundation from USA and the Spanish Ministerio de Ciencia e Innovación (PID2019-106608RB-I00), and the Instituto de Salud Carlos III (CIBERNED, CB18/05/00040). AlM is funded with the Ramon y Cajal program from The Spanish Ministerio de Ciencia e Innovación (RYC-2017-22392).

## Conflict of interest

The authors declare that the research was conducted in the absence of any commercial or financial relationships that could be construed as a potential conflict of interest.

## Publisher's note

All claims expressed in this article are solely those of the authors and do not necessarily represent those of their affiliated organizations, or those of the publisher, the editors and the reviewers. Any product that may be evaluated in this article, or claim that may be made by its manufacturer, is not guaranteed or endorsed by the publisher.
